# Indisulam Shows an Anti-Cancer Effect on HPV+ and HPV− Head and Neck Cancer

**DOI:** 10.3390/cancers17071072

**Published:** 2025-03-22

**Authors:** Tara M. Hosseini, Sophie S. Jang, Joseph Bendik, Theresa Guo

**Affiliations:** 1 Moores Cancer Center, University of California San Diego, La Jolla, CA 92093, USA; 2 Department of Otolaryngology-Head & Neck Surgery, University of California San Diego, La Jolla, CA 92093, USA

**Keywords:** head and neck squamous cell carcinoma, HPV-positive, HPV-negative, HNSCC, indisulam, RBM39, interferon-alpha, interferon-gamma, SCC, HPV

## Abstract

**Background/Objectives**: HPV+ head and neck squamous cell carcinoma has been shown to have a unique genomic background, requiring researchers to study it as its own distinct type of cancer. HPV+ tumors have been shown to exhibit fewer genetic mutations in cancer drivers as opposed to their HPV− counterparts. In this paper, we explored how targeting post-transcriptional changes, specifically alternative splicing events, could serve as a potential mechanism to treat HPV+ cancer. **Methods**: Using indisulam, a drug that targets alternative splicing through the degradation of RBM39, we treated various HPV+ and HPV− cell lines and assessed tumor cell viability. We also tested indisulam in vivo to evaluate its effect on tumor volume. Additionally, we analyzed gene expression differences between indisulam-treated subjects and their non-treated counterparts. **Results**: Indisulam treatment led to a reduction in tumor cell viability in both HPV+ and HPV− cell lines. In vivo experiments showed a reduction in tumor volume following indisulam treatment. Gene expression analysis revealed that indisulam induces consistent differential gene expression changes and highly enriches interferon pathways in treated HPV+ cell lines. **Conclusions**: These findings suggest that targeting alternative splicing via indisulam may be a promising therapeutic approach for HPV+ cancers. Further research is required to establish indisulam as a viable anti-cancer treatment in clinical settings.

## Simple Summary

Head and neck squamous cell carcinoma (HNSCC) is a cancer affecting the mouth, throat, and related regions, with cases linked to smoking, alcohol, or human papillomavirus (HPV) infection. HPV-positive (HPV+) HNSCC differs biologically from HPV-negative (HPV−) cases, requiring unique treatment approaches. This study focuses on indisulam, a drug that degrades a protein called RBM39, to target alternative splicing which is prevalent in HPV+ HNSCC. We found that in HPV+ and HPV− cell lines, indisulam effectively reduces RBM39 levels, decreases cell growth in vitro, and shrinks tumors in vivo. Additionally, it activates anti-tumor immune pathways and impacts gene expression, making it a promising therapeutic candidate. These findings highlight indisulam’s potential to improve treatment outcomes for HNSCC patients, warranting further investigation.

## 1. Introduction

Head and neck squamous cell carcinoma (HNSCC) originates from the epithelium of the upper aerodigestive tract [1]. Traditionally, it has been associated with smoking and/or alcohol consumption; however, there has been a recent increase in oropharynx SCC associated with human papillomavirus (HPV) [1,2]. HNSCC induced by high-risk HPV, specifically HPV-16, has become increasingly prevalent in recent years, with the incident rate predicted to increase significantly by 2030 [3]. Meanwhile, smoking patterns in recent years have decreased the frequency of HPV-negative (HPV−) HNSCCs [3]. Overall, HPV-positive (HPV+) and HPV− HNSCC have varying risk factors and are distinctly different in their response to treatment, suggesting a unique path to tumorigeneses [1,2,4]. HPV+ cancers have a better prognosis than HPV− HNSCC, independent of stage and risk factors like smoking and alcohol use [5,6]. Studies have found that HPV+ and HPV− HNSCC differ in their mutational landscape and gene expression patterns, with HPV+ showing upregulation of cell cycle-related genes and expression of oncogenes E6 and E7 [1,2]. Considering all of this, HPV− and HPV+ cancers are now treated as two distinct types of cancers with modified approaches in their treatment regimens.

HPV+ cancers have a unique biology and genetic landscape when compared to their HPV− counterparts. HPV+ tumors have been shown to exhibit fewer genetic mutations in cancer drivers as opposed to HPV− tumors, driving curiosity towards aberrant post-transcriptional and epigenetic changes that could contribute to the pathogenesis of HPV+ tumors [7,8]. Post-transcriptional changes, specifically alternative splicing events (ASEs), have been suggested as a potential mechanism to explain how carcinogenesis is sustained in HPV+ tumors despite the lack of oncogenic genetic alterations [9]. Previous work has shown that HPV-related oropharynx squamous cell carcinoma has been reported to have a high amount of alternative splicing events, and functionally active splicing variants in AKT3 as well as GSN [10,11]. In targeting these ASEs as part of treatment regimens, it can be proposed to target alternative splicing by reducing and/or disrupting overall splicing events.

Overall splicing events can be targeted by aryl sulfonamide drug indisulam (E7070), which degrades RNA Binding Motif 39 (RBM39), a protein that is essential for transcriptional processes and alternative splicing. Molecularly, indisulam recruits RBM39 to DCAF15, a ubiquitin ligase, and this interaction forms a ternary structure that ultimately results in RBM39 degradation [12,13]. RBM39 plays an indirect role in tumorigenesis through its regulation of gene transcription and alternative splicing of RNA [13]. The loss of RBM39 results in aberrant splicing events and differential gene expression within the tumor microenvironment, and as a result inhibits cell cycle progression and induces anti-tumoral effects [14]. Recent studies have shown indisulam to be an effective anti-cancer agent in several cancer cells such as neuroblastoma, cervical, gastric, and ovarian cancer cells [12,15,16,17].

To date, indisulam’s effect on HNSCC has not been investigated. Herein, we demonstrate indisulam as an effective anti-cancer agent in inhibiting tumor growth, decreasing HNSCC cell viability, and activating interferon alpha and interferon gamma pathways in HPV+ HNSCC.

## 2. Materials and Methods

### 2.1. Cell Viability Assays

Cells were cultured for 96 h under treatment conditions of 1.0 μm indisulam, 0.1 μm indisulam, a vehicle control (DMSO), or media. Cell proliferation was measured at baseline and then at 24 h intervals until 96 h, using 1% AquaBluer (MultiTarget Pharmaceutical, Salt Lake City, UT, USA; Cat. #6001,6015) per manufacture protocol. Fluorescence was measured (540 ex/590 em) by the TECAN Spark microplate reader (TECAN, Mannedorf, Switzerland). Media change for the respective treatment conditions was performed after each cell proliferation assay. Plots were visualized using R package ggplot2 (Version 3.5.1) [18]

### 2.2. Cell Lines

The HPV+ cell lines HMS001, UDSCC2, and UMSCC47 and HPV− cell lines SCC11b, SCC9, FaDu, and NOKSI were graciously provided by Dr. Califano, University of California, San Diego (UCSD), USA. The MOC1 cell line was kindly provided by Dr. Gutkind, UCSD, USA. The HPV− and HPV+ cell lines were cultured in Dulbecco’s Modified Eagle Medium (DMEM) (Gibco, Waltham, MA, USA; Cat. #11995-065) supplemented with 10% fetal bovine serum (FBS) (Gibco, Waltham, MA, USA). SCC9 was cultured in a 1:1 mixture of DMEM and F12 Hams media (Gibco, Waltham, MA, USA; Cat. #11320033) with 10% FBS. MOC1 was cultured in a 2:1 mixture of Iscove’s Modified Dulbecco’s Medium (IMDM) (Cytiva, Marlborough, MA, USA; Cat. #SH30228.FS) and F12 Nutrient Mix (Cytiva, Marlborough, MA, USA; Cat. #SH30026.01) with 5% FBS, 1% penicillin/streptomycin (Gibco, Waltham, MA, USA), 1% amphotericin (Gibco, Waltham, MA, USA), 5 ng/mL Epidermal Growth Factor Human Recombinant (Millipore, Billerica, MA, USA; Cat. #01-107), 400 ng/mL hydrocortisone (Sigma-Aldrich, St. Louis, MO, USA; Cat. #H0135), and 5 mg/mL insulin (Sigma-Aldrich, St. Louis, MO, USA; Cat. #I6634), as described in Judd et al. [19].

### 2.3. Western Blot

Western blot analysis was performed to confirm RBM39 degradation. Cells were lysed in a RIPA buffer and protein quantification was performed with the DC Protein Assay Kit (Bio-Rad, Hercules, CA, USA; Cat. #5000111) per manufacturer protocol. Proteins were separated via electrophoresis using Any kD precast polyacrylamide gel (Bio-Rad, Hercules, CA, USA; Cat. #4569035) and then transferred onto polyvinylidene fluoride membranes. The membrane was blocked in 5% milk in TBS–20% Tween (TBS-T) for one hour at room temperature (RT), followed by primary antibody RBM39 (1:1500, Atlas Antibodies, Bromma, Sweden; Cat. #HPA00159) incubation at 4 °C overnight. The loading control was GAPDH (1:1000 concentration, Cell Signaling Technology, Danvers, MA, USA; Cat. #8884). The membrane was washed in TBS-T 3 times, post primary antibody incubation, and then incubated for 1 h in Anti-Rabbit secondary antibodies (1:10,000, Cell Signaling Technology, Danvers, MA, USA; Cat. #7074) at RT. The membrane was washed an additional 3 times using TBS-T before using an ECL exposing substrate (Thermo Scientific, Waltham, MA, USA; Cat. #32106) to expose the membrane and image the blot on the iBright 1500 (Invitrogen, Waltham, MA, USA).

### 2.4. Q-RT PCR

Primers were designed for RBM39 and obtained from Integrated DNA Technologies, Inc. (IDT, Coralville, IA, USA). The annealing temperature for the primers was 60 °C. The forward primer sequence: GTTCGTCGATGTTAGCTCAGTGC. The reverse primer sequence: AGCCTCATAGGTCCAGCACTTC.

### 2.5. In Vivo

#### 2.5.1. Animals

C57BL/6 mice were obtained from Charles Rivers Lab (Wilmington, MA, USA). All animal studies performed with, supervised, and approved by the IACUC. All murine protocols were approved by the Institutional Animal Care and Use Committee at the University of California, San Diego.

#### 2.5.2. Tumor Establishment and Treatment

Orthotopic oral tumors were established in C57/BL6 male mice (Charles River Laboratories, Wilmington, MA, USA), by injecting 1 × 10^6^ MOC1 cells into the tongue using a 25-gauge insulin syringe. Tumors were measured using calipers every other day. Mice were randomized and administered indisulam (25 mg/kg) or the vehicle control (DMSO) via intraperitoneal injection. Indisulam (Sigma-Aldrich, St. Louis, MO, USA; Cat. #SML1225) was reconstituted in DMSO according to the desired concentration and then diluted further with sterile saline for in vivo injections. Mice were treated once every other day, 6 days post initial tumor establishment, for a total of four injections.

### 2.6. RNA Analysis

#### 2.6.1. RNA Extraction

RNA extraction was performed using Qiagen RNeasy Mini Kits (Qiagen, Venlo, The Netherlands) per manufacturer protocol. RNA quality was assessed via NanoDrop (Thermo Fisher Scientific, Wilmington, DE, USA) and TapeStation (Agilent Technologies, Santa Clara, CA, USA).

#### 2.6.2. RNA Sequencing

RNA was sequenced using NovaSeq S4 (Illumnia, San Diego, CA, USA) by the Institute for Genomic Medicine (IGM) at the University of California, San Diego (UCSD). A PE100 run type was selected, resulting in a mean read depth of 30 million 100 × 100 paired-end reads. Sequencing libraries were generated using a ribodepleted RNA-stranded library.

### 2.7. Computational Analysis

#### 2.7.1. Differential Gene Expression Analysis and Heat Maps

Raw RNA sequences were aligned to both the genome and transcriptome by STAR (Version 2.7.1a). The bam files were provided for the featureCounts function as part of the Rsubread package (Version 2.20.0) in R (Version 4.4.0). to obtain read counts per gene. Multi-mapping reads were excluded from the analysis. These values were given to Deseq2 (Version 1.46.0) for normalization and differential expression [20]. Genes with an average of less than 10 reads were excluded.

HPV+ cell lines HMS001, SCC2, and SCC47 were treated for 4 days with 1.0 μM of indisulam or DMSO and then harvested for RNA on day 4. Raw counts were normalized using DEseq2 [20,21]. After filtering for genes with greater than 10 reads, 29,348 genes were left for analysis. Of these genes, 730 genes were identified to be differentially expressed with a *p*-adjusted value ≤ 0.05. We then conducted unsupervised clustering on these 730 genes. All heat maps were generated using normalized feature counts provided by DEseq2 and the pheatmaps package in R (Version 4.4.0).

#### 2.7.2. GSEA

Normalized DESeq2 data were converted to the GTC file format and then used to run Gene Set Enrichment Analysis (GSEA, Version 4.3.2) [22]. We used annotated Hallmark Gene Sets from the Human MSigDB Collections provided by GSEA to run with the GSEA software [23]. The enriched gene sets reported had a nominal *p*-value < 0.05 and an FDR q-value < 0.05.

#### 2.7.3. Survival and Splicing Analysis

The analysis survival and splicing analysis results were in whole or part based upon data generated by the TCGA Research Network: https://www.cancer.gov/tcga [24]. Kaplan-Meier curves were generated using the survival (Version 3.6.4) and ggsurvfit (Version 1.1.0) packages in R (Version 4.4.1), using survival, splicing, and gene expression HNSCC TCGA data aligned to hg19. The provided splicing data consisted of the number of reads spanning each splicing event. Gene expression data were then obtained through RSEM where the resulting expected read counts per gene underwent quantile normalization [25]. Of the 520 available patient samples with splicing and gene expression data, survival data were available for 463 of these patients. Of these, 457 patients contained gene expression data for the RBM39 gene. From this, a final set of 284 patients with HNSCC, including splicing burden data, gene expression data, survival data, and validated HPV status assignments, was created. In this final set, 41 of the patients were denoted as HPV-positive, and 243 were denoted as HPV-negative [10]. The outlier expression and splicing burden of these tumor samples were then evaluated against 44 normal head and neck tissue samples provided from TCGA data using the Outsplice (Version 1.7.0) package in R [26].

## 3. Results

### 3.1. High Splicing Burden Is Correlated with Higher RBM39 Expression Levels in TCGA HNSCC Data

**Figure 1:**
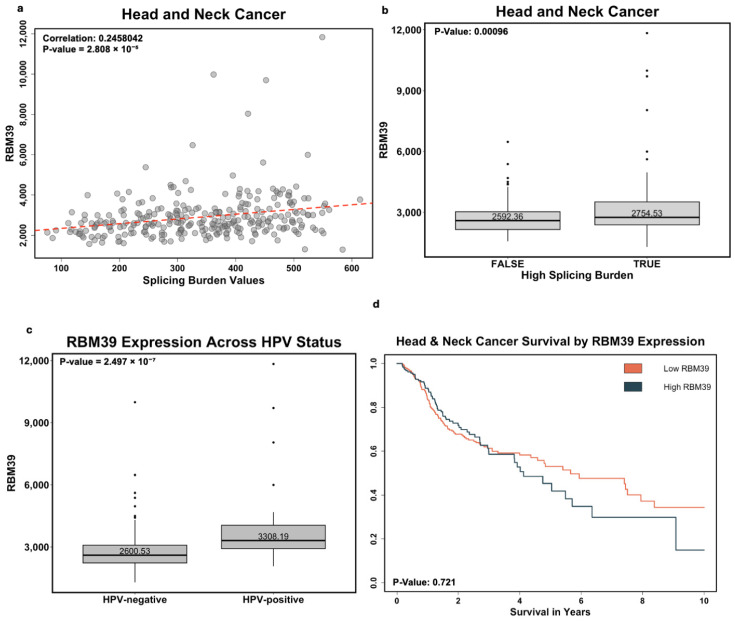
(**a**) Correlation between RBM39 expression and splicing burden in HNSCC. (**b**) Box and whisker plot of RBM39 expression between patients with high and low splicing burden. Expression values are derived from RSEM quantile-normalized expected counts. (**c**) Box and whisker plot of RBM39 expression between HPV+ and HPV− patients. Expression values are derived from RSEM quantile-normalized expected counts. HPV+ *n* = 41. HPV− *n* = 243. (**d**) Kaplan-Meier plot of HNSCC between patients with low or high RBM39 expression.

To evaluate the potential of indisulam to inhibit tumor growth in HNSCC, we first investigated the expression levels of RBM39, a critical target of indisulam, and its relationship with the splicing burden in patient samples. Using TCGA data on HNSCC (Figure 1a), we saw a significant positive correlation between the splicing burden and RBM39 expression, with a correlation value of 0.2458 (*p* = 2.808 × 10^−5^). In Figure 1b, we again show that patients with a high splicing burden had significantly higher expression of RBM39 compared to those with a lower spicing burden (*p* = 0.00096).

**Figure A1:**
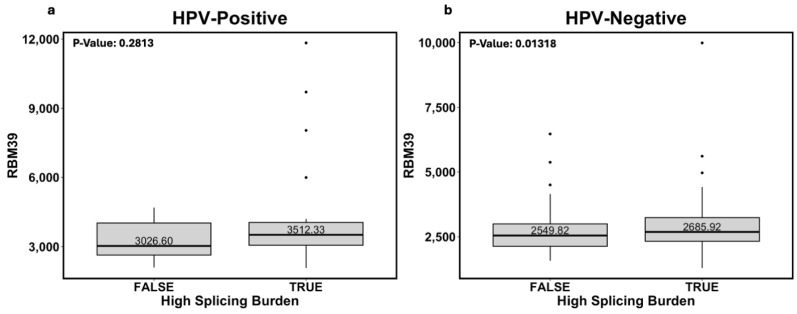
(**a**) Box and whisker plot of RBM39 expression against high and low splicing burden in HPV+ head and neck cancer patients. Expression values are derived from RSEM quantile-normalized expected counts. HPV+ *n* = 41. (**b**) Box and whisker plot of RBM39 expression against high and low splicing burden in HPV− head and neck cancer patients. Expression values are derived from RSEM quantile-normalized expected counts. HPV− *n* = 243.

Next, we evaluated RBM39 expression based on tumor HPV status (Figure 1c). RBM39 expression was significantly elevated in HPV+ tumors compared to HPV− tumors, with RSEM values of 3308.19 and 2600.53, respectively (*p* = 2.497 × 10^−7^). The splicing burden was also higher in HPV+ patients than HPV− patients, with a median of 411 and 342, respectively (*p* = 0.005259). Additionally, when stratifying by HPV status, we observed a significant positive correlation between splicing and RBM39 expression in HPV− patients (*p* = 0.01318); the same trend was seen in HPV+ patients, but did not reach significance, likely due to a limited sample size of HPV+ patients (*n* = 41, Appendix Figure A1a,b).

We also examined RBM39 expression in HNSCC tumor and normal tissue types using RSEM expression values. However, there was no significant difference between the two groups (*p* = 0.1352), with 6.34% of tumor samples having expression two standard deviations above the mean of normal tissues.

Finally, we assessed the impact of RBM39 expression on survival outcomes. Kaplan-Meier analysis suggested that higher RBM39 was associated with lower survival, though this trend did not reach statistical significance (Figure 1d).

These observations suggest that RBM39 is not only correlated to the splicing burden but could also play a role in the progression of HNSCC, particularly in patients with a high splicing burden or HPV+ status. To explore if RBM39 could be a potential therapeutic target, we next examined the effects of indisulam in vitro using HPV+ and HPV− cell lines.

### 3.2. Indisulam Degrades RBM39 and Reducing Cancer Cell Viability in HPV+ and HPV− Cell Lines

**Figure 2:**
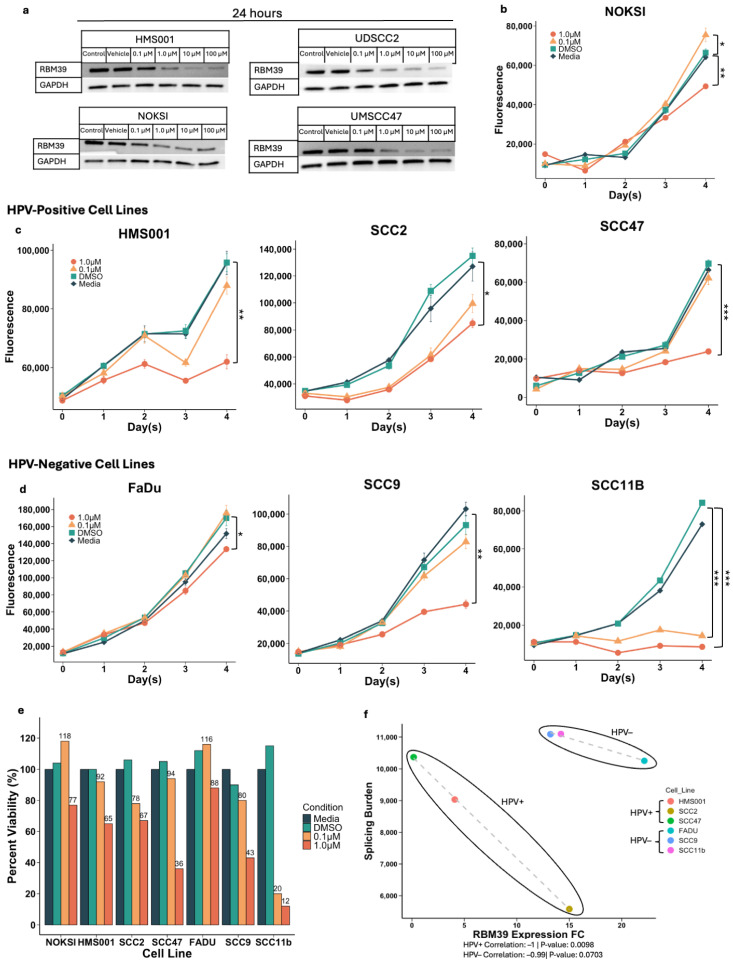
(**a**) Western blot analysis of RBM39 in HPV+ cell lines and NOKSI after 24 h exposure to different doses of indisulam. Uncropped blots are shown in File S1. (**b**) Cell proliferation assay of NOKSI cells with 96 h of 1.0 μM, 0.1 μM, and DMSO treatment. (**c**) Cell proliferation assays of HPV+ cell lines, HMS001, SCC2, and SCC47, with 96 h of 1.0 μM, 0.1 μM, and DMSO treatment. (**d**) Cell proliferation assays of HPV+ cell lines, FaDu, SCC9, and SCC11b, with 96 h of 1.0 μM, 0.1 μM, and DMSO treatment. (**e**) Bar graph with percent viability of each cell line on day 4 after 96 h of 1.0 μM, 0.1 μM, and DMSO treatment. For (**b**–**d**), a *t*-test was used to determine significance. (**f**) Correlation between splicing burden and RBM39 expression in HPV+ and HPV− cell lines. RBM39 expression FC values were determined using QRT-PCR and splicing burden values were derived from OutSplice. * *p* < 0.05, ** *p* < 1.0 × 10^−4^, *** *p* < 1.0 × 10^−7^.

To confirm if indisulam is effective in degrading RBM39 in HPV+ cell lines, cells were incubated with varying concentrations of indisulam for 24 h, followed by protein extraction. Western blot analysis was then conducted on the extracted proteins (Figure 2a). In all the HPV+ cell lines (HMS001, SCC2, SCC47) and the normal keratinocytes control (NOKSI), indisulam treatment was shown to degrade RBM39 protein expression in a dose-dependent manner. Cells were also treated with the same volume of the vehicle control, DMSO, which showed no effect on the RBM39 protein levels.

Following this, we performed a 4-day-long cell proliferation assay to assess indisulam’s effect on HPV+ cancer cell viability. Using a clinically representative dose, cells were treated with 1.0 μM or 0.1 μM doses of indisulam, the vehicle control (DMSO), or media, every day, from day 0 to day 3. Cell viability was measured after each day, from day 0 to day 4. NOKSI as well as all the HPV+ cell lines showed significant differences in cell viability when comparing 1.0 μM indisulam-treated cells to the DMSO and media controls (Figure 2b,c).

We were interested to see if these results would also hold in HPV− HNSCC cell lines. Indisulam treatment also showed reduced cell viability in HPV− cell lines FaDu, SCC9, and SCC11b, with significant differences between control and 1.0 μM indisulam-treated groups. SCC11b also exhibited significantly reduced cell viability at lower indisulam concentrations, with cells treated at 0.1 μM showing viability levels comparable with those in the 1.0 μM experimental group. (Figure 2d). When comparing the day 4 cell viabilities, normalized to their respective media controls (Figure 2e), cell lines had varying degrees of decreased cell viability. Interestingly, all cell lines, including NOKSI, the normal keratinocyte control, showed reductions in cell viability when treated with indisulam, with a day-4 viability of 77% when in the 1.0 μM treated group.

These results imply that indisulam treatment is effective in reducing RBM39 expression and tumor growth in vitro; thus, its therapeutic potential should be tested further in vivo and possibly clinically. With this in mind, we were interested to see if the trends between RBM39 and the splicing burden in HPV+ and HPV− patients would be present in vitro.

Interestingly, when analyzing the correlation between the splicing burden and RBM39 expression in vitro, we saw inverse trends in comparison to our TCGA analysis (Figure 2f). Both HPV+ and HPV− cell lines had a negative correlation between the splicing burden and RBM39 expression, −1 and −0.99, respectively (*p* = 0.0098, *p* = 0.0703). In vitro, the HPV− cell lines had higher levels of RBM39 expression than HPV+ cell lines, and within the HPV+ cell lines, there was greater variation in the splicing burden and RBM39 expression. HPV− cell lines also showed a negative correlation between the splicing burden and growth fold change over time (correlation = −1, *p* = 0.0377), with faster-growing cell lines having a lower splicing burden.

While the trend was not consistent between the patient and in vitro data, this could be attributed to the limitations of in vitro data and the lack of an immune system. To account for this, we sought to test indisulam’s therapeutic potential in vivo.

### 3.3. Indisulam Reduces RBM39 Expression and Cell Proliferation in HPV− Oral Cancer Mouse Model Cell Line MOC1

**Figure 3:**
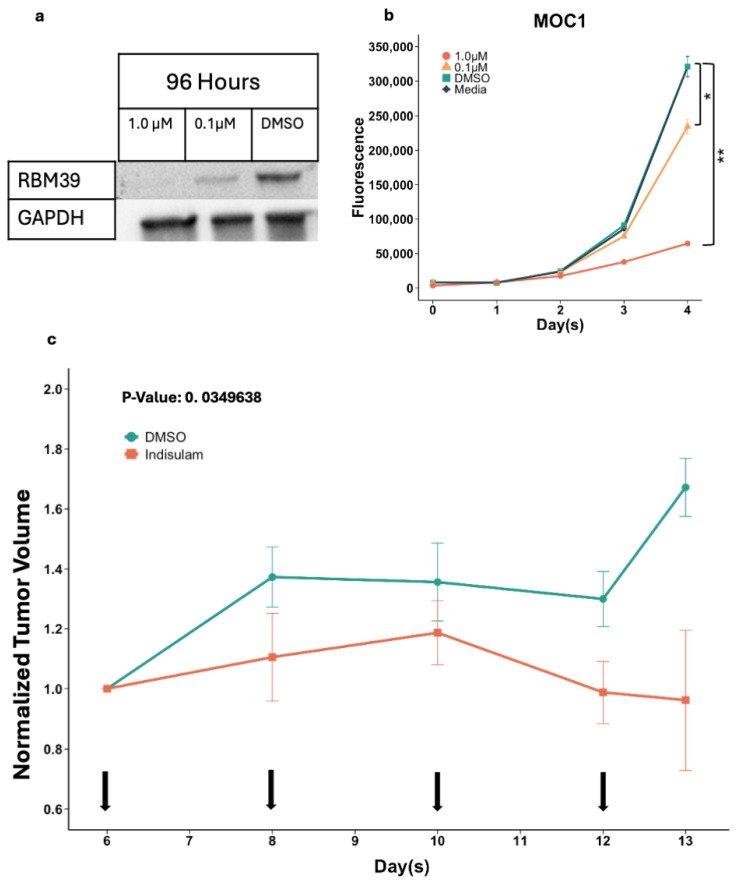
(**a**) Western blot analysis of RBM39 expression in MOC1 cell line after 96 h of treatment with 1.0 μM, 0.1 μM, and DMSO. Uncropped blot is shown in File S1. (**b**) Cell proliferation assay of MOC1 with 96 h of 1.0 μM, 0.1 μM, and DMSO treatment. (**c**) Average tumor volume of MOC1 tongue tumors in DMSO- and indisulam-treated HPV− mice. Arrows indicate injection dates on day 6, 8, 10, and 12. Tumor volume normalized to volume on day 6 prior to injection. * *p* < 0.05, ** *p* < 1.0 × 10^−4^.

Prior to the in vivo implementation, we first assessed the efficacy of indisulam against MOC1 cells in vitro with 1.0 μM and 0.1 μM of indisulam over the course of 96 h (Figure 3a). On the third day, proteins from cells were extracted and Western blot analysis was conducted. Complete degradation of RBM39 was seen when MOC1 cells were treated for three consecutive days with 1.0 μM of indisulam. Partial degradation of RBM39 was seen in the 0.1 μM treated cells when compared to the control DMSO. Following the Western blot, a cell proliferation assay was performed on the MOC1 cells, confirming that the 1.0 μM dose of indisulam decreased cell viability significantly to 20% by the fourth day (Figure 3b). The in vitro experiments with the MOC1 cells confirmed that indisulam was effective in degrading the RBM39 protein and reducing cell viability in both human and murine HNSCC cell lines. We proceeded with the in vivo implementation by generating a murine model of oral cancer using the MOC1 cells.

### 3.4. Indisulam Reduces Tumor Volumes in HPV− Oral Cancer Mouse Model

We generated our in vivo model by injecting 1 million MOC1 cells into the tongues of HPV− C57/BlK6 mice and treating them on days 6, day 8, 10, and day 12 after tumor inoculation with 25 mg/kg of indisulam. Figure 3c displays the average tumor volume of the indisulam-treated and vehicle-treated groups (DMSO), normalized to their respective average day 6 volumes. Within the indisulam-treated group, tumor volume decreased over time, with a significant difference in tumor volume seen on day 13 (*p* = 0.0349638). No significant decreases in weight were observed in the treated mice.

Both our in vitro and in vivo murine oral cancer models showed consistent results in the effectiveness of indisulam as a therapeutic agent. This led us to explore the expressional underpinnings of indisulam treatment on HPV+ and HPV− HNSCC. Aside from degrading RBM39 and disrupting post-transcriptional processes, did indisulam invoke any other type of gene expression changes that aided in its therapeutic functions? To answer this question, we analyzed the differential gene expression between indisulam-treated and untreated HNSCC cell lines.

### 3.5. Indisulam Induces Consistent Differential Gene Expression Changes and Highly Enriches Interferon Pathways in Treated HPV+ Cell Lines

**Figure 4:**
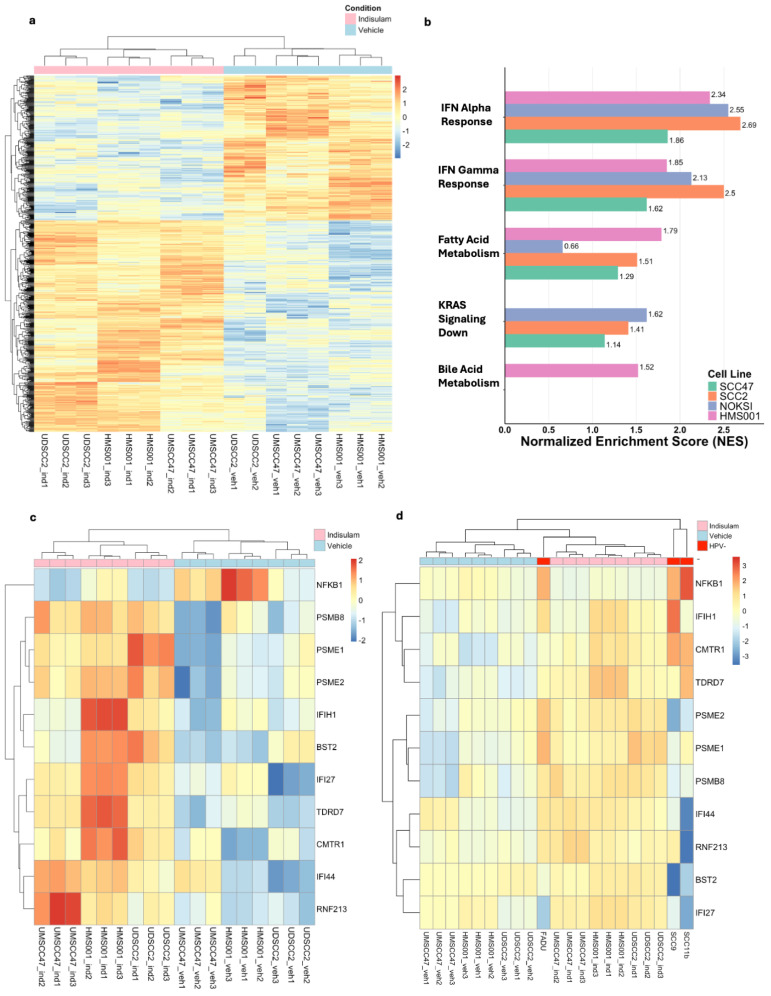
(**a**) Heat map of 730 significantly differentially expressed genes across HPV+ cell lines treated with indisulam, or vehicle (DMSO). (**b**) Bar graph with normalized enrichment scores of gene set pathways from GSEA analysis. (**c**) Heatmap of HPV+ cell lines treated with indisulam with genes that were identified to be both significantly differentially expressed and part of the IFN-α and IFN-γ pathway gene sets. (**d**) Heatmap of HPV+ cell lines treated with indisulam and HPV− untreated cell lines with genes that were previously selected.

We performed a computational analysis to understand the additional mechanisms of how indisulam induces its anti-cancer effects in HNSCC, specifically HPV+ cell lines. Using RNA gathered from our in vitro experiment, 730 genes were identified to be differentially expressed with a *p*-adjusted value ≤ 0.05. We then conducted unsupervised clustering on these 730 genes and found that, despite the cell’s lines varying head and neck cancer origin, samples clustered according to treatment condition, indisulam, or vehicle (Figure 4a). This suggests that across the different HPV+ cell lines, indisulam induced consistent and uniform gene expression changes, with treated samples exhibiting similar alterations regardless of the specific cell line.

We then used GSEA to further analyze what pathways indisulam enriches in the treated cell lines (Figure 4b). The GSEA results showed that across all HPV+ cell lines, the interferon alpha (IFN-α) pathway consistently had the highest normalized enrichment scores (NESs) among the treated samples. Additionally, interferon gamma (IFN-γ) was consistently the second most enriched pathway within the treated samples. After IFN-α and IFN-γ, other pathways that were significantly enriched included fatty acid metabolism, KRAS signaling, and bile acid metabolism. Using the GSEA genes defined in the IFN-α and IFN-γ genes set, we filtered the initial 730 differentially expressed genes and identified 11 genes that were present in both the DESeq2 and GSEA analysis. These genes included NFKB1, PSMB8, PSME2, IFIH1, BST2, IFI27, TDRD7, CMTR1, IFI44, and RNF213 (Figure 4c). When these 11 genes underwent unsupervised clustering, they also clustered based on condition, suggesting that cells upregulate important interferon alpha and gamma pathway genes as a response to indisulam. When HPV− cell lines were included in the heatmap, their expression was most like the indisulam-treated HPV+ samples, as opposed to the HPV+ vehicle samples (Figure 4d). This indicates that indisulam treatment in HPV+ cell lines elevates the expression of these genes, aligning them more closely with the expression levels observed in the untreated HPV− cell lines, rather than their untreated HPV+ counterparts. This alignment was primarily driven by changes in gene expression seen in IFIH1, CMTR1, and TDRD7; however, some genes within this set still showed a different expression pattern in HPV− cell lines compared to indisulam-treated cells, including PSME2, IFI44, and IFI27. Interestingly, the gene NFKB1 showed differential expression between HPV+ and HPV− cell lines, with higher expression in HPV− cell lines but decreased expression in response to indisulam.

We also evaluated RBM39 expression in HPV+ cell lines before and after treatment using RNA-Seq data. Expression values revealed significant decreases in RBM39 levels in indisulam-treated HMS001 and SCC47 samples (*p* = 0.01467 and *p* = 1.45848 × 10^−5^, respectively), consistent with our Western blot findings. SCC2, however, showed no significant reduction (*p* = 0.12448). Importantly, since indisulam primarily degrades RBM39 protein, RNA-Seq data may not fully capture indisulam’s effects, highlighting the necessity of Western blot assays for comprehensive analysis.

## 4. Discussion

RBM39 is overexpressed in many human cancers, including HNSCC, where RBM39 is found to be both highly expressed and negatively correlated with the infiltration of most immune cells [27]. The degradation of RBM39 causes a disruption of splicing, with increased drug concentrations causing increased splicing alterations, and has been demonstrated to suppress tumor growth in vivo across multiple tumor types [28]. With previous work demonstrating the prevalence of ASE in HPV-related oropharynx squamous cell carcinoma, and the limited findings of cancer drivers in HNSCC, we looked at the post-transcriptional systems and effects of targeting splicing in HPV+ HNSCC. Prior to this study, RBM39 expression had not been analyzed between HPV+ and HPV− HNSCC. Our TCGA analysis revealed a positive correlation between high RBM39 expression and an increased splicing burden in HNSCC. Additionally, we also found that HPV+ HNSCC exhibits higher RBM39 expression compared to HPV− HNSCC. Interestingly, these trends were reversed in vitro, with HPV− cell lines having higher RBM39 expression, and HPV+ and HPV− cell lines having a negative correlation between the splicing burden and RBM39. The discrepancies seen between in vitro and human TCGA data may be explained by the impact of the tumor microenvironment which is not present in cell culture models.

RBM39 has been shown to be essential for cancer cell survival both in vitro and in vivo, with the degradation of RBM39 leading to anti-tumoral effects across many cancer cell lines and xenograft models [12,15,28,29,30,31]. Our research also demonstrated that RBM39 degradation was correlated with the inhibition of cancer cell growth, supporting RBM39’s essential role in the growth of cancer cells. In HPV+ cell lines, RBM39 expression decreased as the indisulam dose concentration increased, an effect also observed in acute myeloid leukemia, cervical, neuroblastoma, and other cancer cell lines [12,15,28,30,31].

In our study treating HPV+ cells with indisulam, we found that indisulam significantly inhibited the growth of both HPV+ and HPV− cancer cells. Additionally, in vivo experiments revealed that indisulam-treated tumors had suppressed tumor growth. These results were also reciprocated in Dou et al., where indisulam was used to treat human HPV+ cervical cancer cells and in vivo xenografts [15]. Dou et al. found that indisulam exerted anti-cancer effects both in vitro and in vivo, inhibiting the growth of cervical cancer cells in the culture in a dose-dependent fashion and suppressing tumor growth [15]. Other studies have also shown that xenograft in vivo cancer models respond to indisulam, including cervical, neuroblastoma, and acute myeloid leukemia [12,15,30,31].

Previous studies with indisulam, conducted by Lu et al., have demonstrated that the splicing modulation induced by RBM39 degradation generated neoantigens that could be recognized by immune cells, triggering an anti-tumor T-cell response and the inhibition of tumor growth [28]. This belief that the alteration of RNA splicing has an immunomodulatory effect that can enhance anti-cancer effects was also reflected in our study. The degradation of RBM39 in the HPV+ cell lines coupled with decreased cell viability and tumor volume in our pre-clinical models shows that indisulam also shows anti-cancer effects in HPV+ and HPV− HNSCC. In addition, our GSEA analysis demonstrated that indisulam-treated groups experience a significant enrichment in the pathways of IFN-α and IFN-γ relative to their untreated counterparts, a finding that has not been previously noted. In addition, the differential gene expression changes within the IFN-α and IFN-γ pathways are different across HPV status, with indisulam-treated HPV+ samples’ gene expression being more similar to untreated HPV− samples than vehicle-treated HPV+ samples.

IFN-α and IFN-γ are cytokines produced by the innate immune system in response to viruses [32]. HPV targets and evades this response by deregulating the various components of the IFN pathway through the molecular actions of oncoproteins, such as E1, E2, E5, E6, and E7, which alter interferon production and prevent interferon-stimulated gene pathway activation [32]. Both IFN-α and IFN-γ have been reported to decrease the amount of HPV-18 E6 and E7 transcripts in HeLa cells, and high-dose IFN-α has been utilized as a form of adjuvant therapy to treat melanoma as well as to clear subclinical and latent HPV infections [33,34,35]. IFN-γ has also shown efficacy in reducing the rate of HPV-16 infections by inhibiting viral entry into the host cell and thus preventing viral transcription and translation [36].

Among the genes highlighted in our analysis of the IFN-α and IFN-γ pathways were interferon genes IFIH1, IFI27, and IFI44. These genes were consistently upregulated in indisulam-treated HPV+ cell lines and have been reported to have connections with various HPV+ cancers [37,38,39]. IFI44 was specifically studied in HNSCC and reported to have prognostic value, with overexpression of these genes correlated with worse clinical outcomes [38]. In Pan et al., immune signature analysis of IFI44 expression showed a positive correlation between IFI44 expression and CD4+ cells, macrophages, and neutrophils in HNSCC [38]. They also looked at HPV+ oropharyngeal squamous cell carcinoma, which had significantly higher IFI44 expression than normal cancer [38].

## 5. Conclusions

In conclusion, our study highlights the potential of indisulam as a therapeutic agent in treating HPV+-related head and neck cancer. The findings highlight that indisulam is effective in targeting the critical processes of cancer growth and progression. A limitation of the current study is that the experiments were conducted primarily in vitro, meaning that the interactions with the immune system and other systemic effects should be further explored. In future studies, indisulam could be considered as a novel therapeutic target in HPV+ HNSCC as part of a combinatory treatment; this would be imperative to understand its full anti-tumoral potential and clinical translation ability.

## Data Availability

Sequencing data for cell line and mouse experiments are available upon request.

## Supplementary Materials

The following supporting information can be downloaded at: https://www.mdpi.com/article/10.3390/cancers17071072/s1, File S1: Uncropped western blots.

## Abbreviations

The following abbreviations are used in this manuscript:

HPV	Human papillomavirus
HNSCC	Head and neck squamous cell carcinoma
ASE	Alternative splicing event
