# Supplementary figures and images for: Indisulam Shows an Anti-Cancer Effect on HPV+ and HPV− Head and Neck Cancer
Source: Cancers (Basel). 2025 Mar 22;17(7):1072. doi: 10.3390/cancers17071072 (PMC11987906; doi:10.3390/cancers17071072)

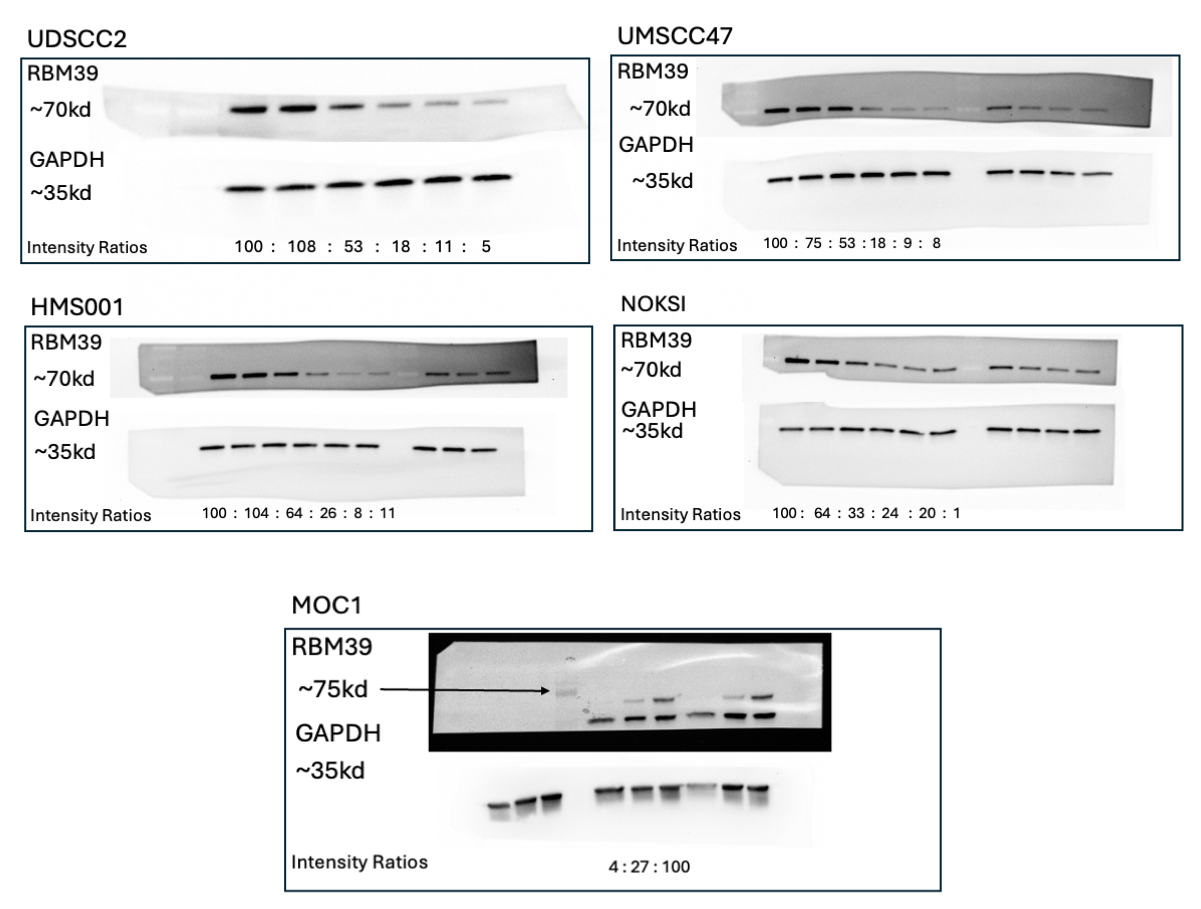

Supplement: Supplementary file 1 [file cancers-17-01072-s001.zip › File S1. Uncropped western blots.tiff]
